# Supplementary material for: The genome sequence of Eledona agricola (Herbst, 1783) (Coleoptera: Tenebrionidae)
Source: Wellcome Open Res. 2025 Sep 9;10:501. [Version 1] doi: 10.12688/wellcomeopenres.24809.1 (PMC12686787; doi:10.12688/wellcomeopenres.24809.1)
Supplement: Peer review history

# Reviewer report: Reviewer response for version 1

**DOI:** 10.21956/wellcomeopenres.27332.r137905

**Published:** 2025-12-23

**Authors:** Nikoletta Andrea Nagy

**Affiliations:** 1 University of Debrecen, Debrecen, Hungary

The manuscript is well-written and technically sound. I have a few comments:

 - "he suggestion that this beetle occurs in the Nearctic..." - missing "T" at the beginning of the sentence

 - species scientific names should be italicized in the methods

 - please include the sex of the specimen used for RNA sequencing

Are sufficient details of methods and materials provided to allow replication by others?

Yes

Is the rationale for creating the dataset(s) clearly described?

Yes

Are the datasets clearly presented in a useable and accessible format?

Yes

Are the protocols appropriate and is the work technically sound?

Yes

Reviewer Expertise:

arthropod genomics

I confirm that I have read this submission and believe that I have an appropriate level of expertise to confirm that it is of an acceptable scientific standard.

# Reviewer report: Reviewer response for version 1

**DOI:** 10.21956/wellcomeopenres.27332.r137906

**Published:** 2025-12-08

**Authors:** Matjaž Gregorič

**Affiliations:** 1 Jovan Hadži Institute of Biology, Research Centre of the Slovenian Academy of Sciences and Arts, Ljubljana, Slovenia

This genome report on the tenebrionid beetle *Eledona agricola* (Herbst, 1783) is thoroughly written and I have no concerns regarding its indexing. There a few typos but I can’t mark them in text: “he” to “The” in the third paragraph, and Eledona agricola and Laetiporus sulphureus need to be italicized at the beginning of the Methods.

Are sufficient details of methods and materials provided to allow replication by others?

Yes

Is the rationale for creating the dataset(s) clearly described?

Yes

Are the datasets clearly presented in a useable and accessible format?

Yes

Are the protocols appropriate and is the work technically sound?

Yes

Reviewer Expertise:

Systematics and taxonomy, evolutionary biology.

I confirm that I have read this submission and believe that I have an appropriate level of expertise to confirm that it is of an acceptable scientific standard.

# Reviewer report: Reviewer response for version 1

**DOI:** 10.21956/wellcomeopenres.27332.r137904

**Published:** 2025-11-25

**Authors:** João Pedro Marques

**Affiliations:** 1 BIOPOLIS-CIBIO, Portugal, Portugal

I read the manuscript *“The genome sequence of Eledona agricola” *carefully *.* In my opinion, he genome was produced following the current best practices in reference genome generation, fully consistent with the standards adopted by large-scale initiatives such as the Darwin Tree of Life project.

 The authors provide a clear and comprehensive description of specimen processing, barcoding verification, high-molecular-weight DNA extraction, PacBio HiFi sequencing, Hi-C scaffolding, RNA-seq generation, and extensive quality control. Assembly statistics (e.g., scaffold N50 of 22.63 Mb, BUSCO completeness of 99.5%, QV ~ 63) fall well within, and in several aspects exceed, accepted benchmarks for high-quality chromosome-level assemblies. Figures and tables are clear, and the dataset is accessible through the ENA. No methodological weaknesses or analytical shortcomings were identified.

 Importantly, the authors report no steps that raise scientific concerns, and all protocols (sequencing, assembly, curation, and contamination screening) follow validated pipelines and softwares (e.g., Hifiasm, YaHS, purge_dups, BlobToolKit, Merqury). The manuscript is consistent and scientifically correct.

 I do not have any requests for revision. In my opinion, the manuscript meets all criteria for publication in its current form.

Are sufficient details of methods and materials provided to allow replication by others?

Yes

Is the rationale for creating the dataset(s) clearly described?

Yes

Are the datasets clearly presented in a useable and accessible format?

Yes

Are the protocols appropriate and is the work technically sound?

Yes

Reviewer Expertise:

Evolutionary Biology, Population Genomics and Genome Assembly

I confirm that I have read this submission and believe that I have an appropriate level of expertise to confirm that it is of an acceptable scientific standard.
